# 3-Cyclo­hexyl­sulfonyl-5-fluoro-2-methyl-1-benzofuran

**DOI:** 10.1107/S1600536811008580

**Published:** 2011-03-12

**Authors:** Hong Dae Choi, Pil Ja Seo, Byeng Wha Son, Uk Lee

**Affiliations:** a Department of Chemistry, Dongeui University, San 24 Kaya-dong Busanjin-gu, Busan 614-714, Republic of Korea; b Department of Chemistry, Pukyong National University, 599-1 Daeyeon 3-dong, Nam-gu, Busan 608-737, Republic of Korea

## Abstract

In the title compound, C_15_H_17_FO_3_S, the cyclo­hexyl ring adopts a classic chair conformation and the aryl­sulfonyl unit is positioned equatorial relative to the cyclo­hexyl group. In the crystal, mol­ecules are linked through weak inter­molecular C—H⋯O hydrogen bonds.

## Related literature

For the pharmacological activity of benzofuran compounds, see: Aslam *et al.* (2006 ▶); Galal *et al.* (2009 ▶); Khan *et al.* (2005 ▶). For natural products with benzofuran rings, see: Akgul & Anil (2003 ▶); Soekamto *et al.* (2003 ▶). For structural studies of related 3-aryl­sulfonyl-5-fluoro-2-methyl-1-benzofuran derivatives, see: Choi *et al.* (2010**a* ▶,b* ▶). 
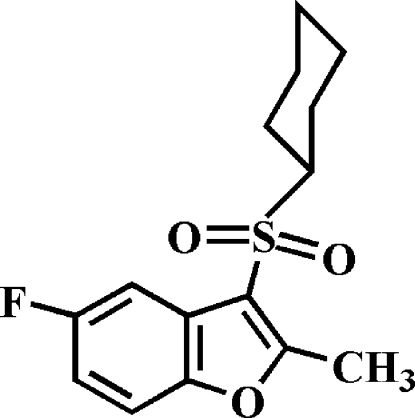


## Experimental

### 

#### Crystal data

C_15_H_17_FO_3_S
*M* *_r_* = 296.35
Monoclinic, 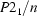

*a* = 5.5830 (4) Å
*b* = 26.879 (2) Å
*c* = 9.1092 (7) Å
β = 93.762 (1)°
*V* = 1364.04 (18) Å^3^
*Z* = 4
Mo *K*α radiation
μ = 0.25 mm^−1^
*T* = 173 K
0.23 × 0.16 × 0.14 mm

#### Data collection

Bruker SMART APEXII CCD diffractometer
Absorption correction: multi-scan (*SADABS*; Bruker, 2009 ▶) *T*_min_ = 0.654, *T*_max_ = 0.746
12713 measured reflections
3141 independent reflections
2682 reflections with *I* > 2σ(*I*)
*R*_int_ = 0.035

#### Refinement

*R*[*F* ^2^ > 2σ(*F* ^2^)] = 0.041
*wR*(*F* ^2^) = 0.106
*S* = 1.05
3141 reflections
182 parameters
H-atom parameters constrained
Δρ_max_ = 0.52 e Å^−3^
Δρ_min_ = −0.38 e Å^−3^

### 

Data collection: *APEX2* (Bruker, 2009 ▶); cell refinement: *SAINT* (Bruker, 2009 ▶); data reduction: *SAINT*; program(s) used to solve structure: *SHELXS97* (Sheldrick, 2008 ▶); program(s) used to refine structure: *SHELXL97* (Sheldrick, 2008 ▶); molecular graphics: *ORTEP-3* (Farrugia, 1997 ▶) and *DIAMOND* (Brandenburg, 1998 ▶); software used to prepare material for publication: *SHELXL97*.

## Supplementary Material

Crystal structure: contains datablocks global, I. DOI: 10.1107/S1600536811008580/jj2079sup1.cif

Structure factors: contains datablocks I. DOI: 10.1107/S1600536811008580/jj2079Isup2.hkl

Additional supplementary materials:  crystallographic information; 3D view; checkCIF report

## Figures and Tables

**Table 1:** Hydrogen-bond geometry (Å, °)

*D*—H⋯*A*	*D*—H	H⋯*A*	*D*⋯*A*	*D*—H⋯*A*
C6—H6⋯O2^i^	0.95	2.44	3.306 (2)	151
C10—H10⋯O3^ii^	1.00	2.33	3.274 (2)	157

Symmetry codes: (i) 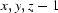; (ii) 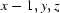.

**Fig. 1.**
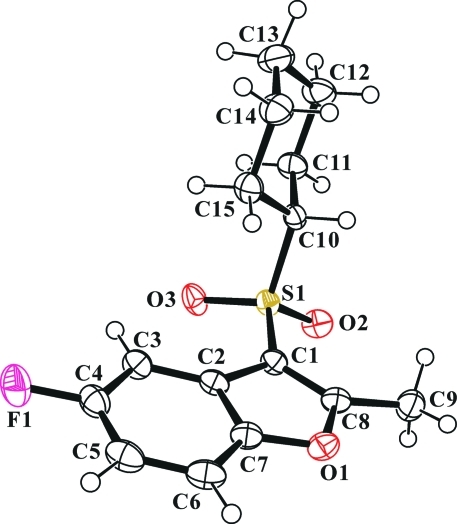
The molecular structure of the title compound with the atom numbering scheme. Displacement ellipsoids are drawn at the 50% probability level. H atoms are presented as a small spheres of arbitrary radius.

**Fig. 2.**
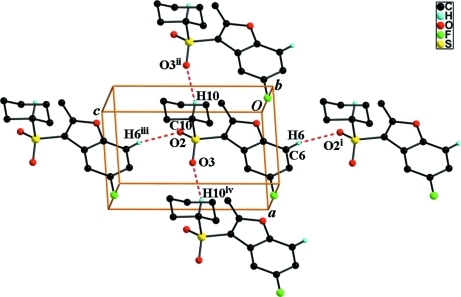
A view of the C–H···O hydrogen bonds (dotted lines) in the crystal structure of the title compound. [Symmetry codes: (i) x, y, z - 1; (ii) x - 1, y, z; (iii) x, y, z + 1; (iv) x + 1, y, z.]

## supplementary crystallographic information

### Comment

Many compounds having a benzofuran ring system exhibit diverse pharmacological properties such as antifungal, antitumor and antiviral, and antimicrobial activities (Aslam *et al.*, 2006, Galal *et al.*, 2009, Khan *et al.*, 2005). These compounds occur in a wide range of natural products (Akgul & Anil, 2003; Soekamto *et al.*, 2003). As a part of our ongoing study of the substituent effect on the solid state structures of 3-arylsulfonyl-5-fluoro-2-methyl-1-benzofuran analogues (Choi *et al.*, 2010**a*,b*), we report herein the crystal structure of the title compound.

In the title molecule (Fig. 1), the benzofuran unit is essentially planar, with a mean deviation of 0.008 (1) Å from the least-squares plane defined by the nine constituent atoms. The cyclohexyl ring is in the chair form and arylsulfonyl moiety is positioned equatorial relative to the cyclohexyl group. The molecular packing (Fig. 2) is stabilized by weak intermolecular C–H···O hydrogen bonds; the first one between a benzene H atom and the O atom of the sulfonyl group (Table 1; C6–H6···O2^i^), and the second one between a cyclohexyl H atom and the O atom of the sulfonyl group (Table 1; C10–H10···O3^ii^).

### Experimental

77% 3-chloroperoxybenzoic acid (560 mg, 2.5 mmol) was added in small portions to a stirred solution of 3-cyclohexylsulfanyl-5-fluoro-2-methyl-1-benzofuran (317 mg, 1.2 mmol) in dichloromethane (40 mL) at 273 K. After being stirred at room temperature for 5h, the mixture was washed with saturated sodium bicarbonate solution and the organic layer was separated, dried over magnesium sulfate, filtered and concentrated at reduced pressure. The residue was purified by column chromatography (hexane-ethyl acetate, 4:1 v/v) to afford the title compound as a colorless solid [yield 73%, m.p. 411-412 K; *R*_f_ = 0.71 (hexane-ethyl acetate, 4:1 v/v)]. Single crystals suitable for X-ray diffraction were prepared by slow evaporation of a solution of the title compound in acetone at room temperature.

### Refinement

All H atoms were positioned geometrically and refined using a riding model, with C–H = 0.95 Å  for aryl, 1.00 Å   for methine, 0.99 Å   for methylene and 0.98 Å   for methyl H atoms, respectively. *U*_iso_(H) =1.2*U*_eq_(C)  for aryl,  methine and methylene, and 1.5*U*_eq_(C) for methyl H atoms.

### Crystal data

**Table:**

C_15_H_17_FO_3_S	*F*(000) = 624
*M**_r_* = 296.35	*D*_x_ = 1.443 Mg m^−^^3^
Monoclinic, *P*2_1_/*n*	Mo *K*α radiation, λ = 0.71073 Å
Hall symbol: -P 2yn	Cell parameters from 4637 reflections
*a* = 5.5830 (4) Å	θ = 2.4–27.5°
*b* = 26.879 (2) Å	µ = 0.25 mm^−^^1^
*c* = 9.1092 (7) Å	*T* = 173 K
β = 93.762 (1)°	Block, colourless
*V* = 1364.04 (18) Å^3^	0.23 × 0.16 × 0.14 mm
*Z* = 4	

### Data collection

**Table:**

Bruker SMART APEXII CCD diffractometer	3141 independent reflections
Radiation source: rotating anode	2682 reflections with *I* > 2σ(*I*)
graphite multilayer	*R*_int_ = 0.035
Detector resolution: 10.0 pixels mm^-1^	θ_max_ = 27.5°, θ_min_ = 1.5°
φ and ω scans	*h* = −7→7
Absorption correction: multi-scan (*SADABS*; Bruker, 2009)	*k* = −34→34
*T*_min_ = 0.654, *T*_max_ = 0.746	*l* = −11→11
12713 measured reflections	

### Refinement

**Table:**

Refinement on *F*^2^	Primary atom site location: structure-invariant direct methods
Least-squares matrix: full	Secondary atom site location: difference Fourier map
*R*[*F*^2^ > 2σ(*F*^2^)] = 0.041	Hydrogen site location: difference Fourier map
*wR*(*F*^2^) = 0.106	H-atom parameters constrained
*S* = 1.05	*w* = 1/[σ^2^(*F*_o_^2^) + (0.046*P*)^2^ + 0.8694*P*] where *P* = (*F*_o_^2^ + 2*F*_c_^2^)/3
3141 reflections	(Δ/σ)_max_ = 0.001
182 parameters	Δρ_max_ = 0.52 e Å^−^^3^
0 restraints	Δρ_min_ = −0.38 e Å^−^^3^

### Special details

**Table:**

Geometry. All esds (except the esd in the dihedral angle between two l.s. planes) are estimated using the full covariance matrix. The cell esds are taken into account individually in the estimation of esds in distances, angles and torsion angles; correlations between esds in cell parameters are only used when they are defined by crystal symmetry. An approximate (isotropic) treatment of cell esds is used for estimating esds involving l.s. planes.
Refinement. Refinement of F^2^ against ALL reflections. The weighted R-factor wR and goodness of fit S are based on F^2^, conventional R-factors R are based on F, with F set to zero for negative F^2^. The threshold expression of F^2^ > 2sigma(F^2^) is used only for calculating R-factors(gt) etc. and is not relevant to the choice of reflections for refinement. R-factors based on F^2^ are statistically about twice as large as those based on F, and R- factors based on ALL data will be even larger.

### Fractional atomic coordinates and isotropic or equivalent isotropic displacement parameters (Å^2^)

**Table:**

	*x*	*y*	*z*	*U*_iso_*/*U*_eq_	
S1	0.43863 (7)	0.591832 (16)	0.47491 (4)	0.02169 (13)	
F1	1.0284 (2)	0.65629 (5)	0.01106 (14)	0.0425 (3)	
O1	0.2604 (2)	0.53800 (5)	0.08250 (13)	0.0277 (3)	
O2	0.3535 (2)	0.55223 (5)	0.56384 (14)	0.0301 (3)	
O3	0.6834 (2)	0.60843 (5)	0.50319 (14)	0.0305 (3)	
C1	0.4053 (3)	0.57456 (6)	0.29077 (18)	0.0223 (3)	
C2	0.5493 (3)	0.59124 (6)	0.17416 (18)	0.0229 (3)	
C3	0.7474 (3)	0.62235 (7)	0.1628 (2)	0.0265 (4)	
H3	0.8195	0.6395	0.2457	0.032*	
C4	0.8324 (3)	0.62684 (7)	0.0254 (2)	0.0304 (4)	
C5	0.7336 (4)	0.60331 (8)	−0.0995 (2)	0.0335 (4)	
H5	0.8002	0.6083	−0.1917	0.040*	
C6	0.5370 (4)	0.57253 (7)	−0.0889 (2)	0.0310 (4)	
H6	0.4646	0.5558	−0.1723	0.037*	
C7	0.4519 (3)	0.56741 (6)	0.04852 (19)	0.0256 (4)	
C8	0.2371 (3)	0.54272 (6)	0.23030 (19)	0.0249 (4)	
C9	0.0458 (4)	0.51238 (7)	0.2908 (2)	0.0324 (4)	
H9A	0.1155	0.4817	0.3334	0.049*	
H9B	−0.0752	0.5040	0.2119	0.049*	
H9C	−0.0296	0.5313	0.3673	0.049*	
C10	0.2431 (3)	0.64368 (6)	0.49108 (18)	0.0217 (3)	
H10	0.0757	0.6320	0.4648	0.026*	
C11	0.2542 (4)	0.66111 (7)	0.6511 (2)	0.0288 (4)	
H11A	0.2134	0.6332	0.7157	0.035*	
H11B	0.4190	0.6723	0.6813	0.035*	
C12	0.0778 (4)	0.70386 (7)	0.6677 (2)	0.0356 (5)	
H12A	−0.0881	0.6916	0.6464	0.043*	
H12B	0.0916	0.7160	0.7705	0.043*	
C13	0.1256 (4)	0.74662 (8)	0.5641 (2)	0.0397 (5)	
H13A	0.0023	0.7727	0.5732	0.048*	
H13B	0.2844	0.7614	0.5924	0.048*	
C14	0.1207 (4)	0.72886 (7)	0.4055 (2)	0.0349 (4)	
H14A	0.1623	0.7569	0.3415	0.042*	
H14B	−0.0436	0.7177	0.3739	0.042*	
C15	0.2966 (3)	0.68617 (7)	0.3869 (2)	0.0291 (4)	
H15A	0.2820	0.6741	0.2840	0.035*	
H15B	0.4630	0.6981	0.4086	0.035*	

### Atomic displacement parameters (Å^2^)

**Table:**

	*U*^11^	*U*^22^	*U*^33^	*U*^12^	*U*^13^	*U*^23^
S1	0.0208 (2)	0.0277 (2)	0.0166 (2)	−0.00039 (16)	0.00182 (15)	−0.00055 (15)
F1	0.0383 (7)	0.0517 (8)	0.0391 (7)	−0.0079 (6)	0.0143 (6)	0.0069 (6)
O1	0.0329 (7)	0.0283 (7)	0.0215 (6)	−0.0016 (5)	−0.0005 (5)	−0.0035 (5)
O2	0.0399 (7)	0.0296 (7)	0.0212 (6)	−0.0001 (6)	0.0053 (6)	0.0035 (5)
O3	0.0188 (6)	0.0474 (8)	0.0251 (6)	−0.0011 (6)	0.0000 (5)	−0.0050 (6)
C1	0.0234 (8)	0.0253 (8)	0.0183 (8)	0.0014 (7)	0.0018 (7)	−0.0005 (6)
C2	0.0253 (8)	0.0250 (8)	0.0184 (8)	0.0054 (7)	0.0022 (7)	0.0008 (6)
C3	0.0268 (9)	0.0295 (9)	0.0232 (9)	0.0016 (7)	0.0022 (7)	0.0010 (7)
C4	0.0297 (9)	0.0318 (10)	0.0307 (10)	0.0031 (8)	0.0085 (8)	0.0061 (7)
C5	0.0421 (11)	0.0375 (10)	0.0218 (9)	0.0089 (9)	0.0096 (8)	0.0041 (8)
C6	0.0421 (11)	0.0324 (10)	0.0184 (8)	0.0081 (8)	0.0013 (8)	−0.0012 (7)
C7	0.0296 (9)	0.0244 (8)	0.0228 (9)	0.0044 (7)	0.0009 (7)	0.0003 (7)
C8	0.0280 (9)	0.0246 (8)	0.0219 (8)	0.0031 (7)	0.0017 (7)	−0.0012 (6)
C9	0.0324 (10)	0.0323 (10)	0.0326 (10)	−0.0060 (8)	0.0036 (8)	−0.0016 (8)
C10	0.0187 (7)	0.0258 (8)	0.0208 (8)	−0.0012 (7)	0.0036 (6)	−0.0013 (6)
C11	0.0336 (10)	0.0318 (9)	0.0215 (8)	0.0018 (8)	0.0056 (7)	−0.0019 (7)
C12	0.0421 (11)	0.0352 (10)	0.0307 (10)	0.0035 (9)	0.0102 (9)	−0.0065 (8)
C13	0.0495 (13)	0.0268 (10)	0.0433 (12)	0.0015 (9)	0.0070 (10)	−0.0050 (8)
C14	0.0401 (11)	0.0285 (9)	0.0366 (11)	0.0019 (8)	0.0058 (9)	0.0055 (8)
C15	0.0312 (9)	0.0311 (9)	0.0257 (9)	−0.0013 (8)	0.0068 (8)	0.0036 (7)

### Geometric parameters (Å, °)

**Table:**

S1—O2	1.4373 (13)	C9—H9B	0.9800
S1—O3	1.4444 (13)	C9—H9C	0.9800
S1—C1	1.7386 (17)	C10—C15	1.527 (2)
S1—C10	1.7824 (17)	C10—C11	1.529 (2)
F1—C4	1.364 (2)	C10—H10	1.0000
O1—C8	1.367 (2)	C11—C12	1.527 (3)
O1—C7	1.381 (2)	C11—H11A	0.9900
C1—C8	1.360 (2)	C11—H11B	0.9900
C1—C2	1.445 (2)	C12—C13	1.521 (3)
C2—C7	1.391 (2)	C12—H12A	0.9900
C2—C3	1.396 (2)	C12—H12B	0.9900
C3—C4	1.373 (3)	C13—C14	1.521 (3)
C3—H3	0.9500	C13—H13A	0.9900
C4—C5	1.384 (3)	C13—H13B	0.9900
C5—C6	1.383 (3)	C14—C15	1.527 (3)
C5—H5	0.9500	C14—H14A	0.9900
C6—C7	1.375 (2)	C14—H14B	0.9900
C6—H6	0.9500	C15—H15A	0.9900
C8—C9	1.479 (3)	C15—H15B	0.9900
C9—H9A	0.9800		
			
O2—S1—O3	118.18 (8)	C15—C10—C11	111.49 (15)
O2—S1—C1	109.13 (8)	C15—C10—S1	112.80 (12)
O3—S1—C1	107.13 (8)	C11—C10—S1	109.37 (12)
O2—S1—C10	107.78 (8)	C15—C10—H10	107.7
O3—S1—C10	108.73 (8)	C11—C10—H10	107.7
C1—S1—C10	105.14 (8)	S1—C10—H10	107.7
C8—O1—C7	107.02 (13)	C12—C11—C10	109.78 (15)
C8—C1—C2	107.46 (15)	C12—C11—H11A	109.7
C8—C1—S1	125.88 (13)	C10—C11—H11A	109.7
C2—C1—S1	126.66 (13)	C12—C11—H11B	109.7
C7—C2—C3	118.95 (16)	C10—C11—H11B	109.7
C7—C2—C1	104.82 (15)	H11A—C11—H11B	108.2
C3—C2—C1	136.22 (17)	C13—C12—C11	111.51 (16)
C4—C3—C2	116.29 (17)	C13—C12—H12A	109.3
C4—C3—H3	121.9	C11—C12—H12A	109.3
C2—C3—H3	121.9	C13—C12—H12B	109.3
F1—C4—C3	117.75 (18)	C11—C12—H12B	109.3
F1—C4—C5	117.76 (17)	H12A—C12—H12B	108.0
C3—C4—C5	124.49 (18)	C14—C13—C12	111.04 (16)
C6—C5—C4	119.45 (17)	C14—C13—H13A	109.4
C6—C5—H5	120.3	C12—C13—H13A	109.4
C4—C5—H5	120.3	C14—C13—H13B	109.4
C7—C6—C5	116.54 (18)	C12—C13—H13B	109.4
C7—C6—H6	121.7	H13A—C13—H13B	108.0
C5—C6—H6	121.7	C13—C14—C15	111.70 (17)
C6—C7—O1	125.48 (17)	C13—C14—H14A	109.3
C6—C7—C2	124.28 (18)	C15—C14—H14A	109.3
O1—C7—C2	110.23 (15)	C13—C14—H14B	109.3
C1—C8—O1	110.46 (15)	C15—C14—H14B	109.3
C1—C8—C9	133.81 (17)	H14A—C14—H14B	107.9
O1—C8—C9	115.71 (16)	C10—C15—C14	109.86 (15)
C8—C9—H9A	109.5	C10—C15—H15A	109.7
C8—C9—H9B	109.5	C14—C15—H15A	109.7
H9A—C9—H9B	109.5	C10—C15—H15B	109.7
C8—C9—H9C	109.5	C14—C15—H15B	109.7
H9A—C9—H9C	109.5	H15A—C15—H15B	108.2
H9B—C9—H9C	109.5		
			
O2—S1—C1—C8	28.15 (18)	C3—C2—C7—O1	179.22 (15)
O3—S1—C1—C8	157.20 (16)	C1—C2—C7—O1	0.18 (19)
C10—S1—C1—C8	−87.23 (17)	C2—C1—C8—O1	−0.6 (2)
O2—S1—C1—C2	−151.98 (15)	S1—C1—C8—O1	179.25 (12)
O3—S1—C1—C2	−22.93 (18)	C2—C1—C8—C9	177.52 (19)
C10—S1—C1—C2	92.64 (16)	S1—C1—C8—C9	−2.6 (3)
C8—C1—C2—C7	0.27 (19)	C7—O1—C8—C1	0.75 (19)
S1—C1—C2—C7	−179.62 (13)	C7—O1—C8—C9	−177.78 (15)
C8—C1—C2—C3	−178.5 (2)	O2—S1—C10—C15	−173.03 (13)
S1—C1—C2—C3	1.6 (3)	O3—S1—C10—C15	57.74 (15)
C7—C2—C3—C4	−0.2 (3)	C1—S1—C10—C15	−56.72 (14)
C1—C2—C3—C4	178.45 (19)	O2—S1—C10—C11	62.29 (14)
C2—C3—C4—F1	−178.89 (16)	O3—S1—C10—C11	−66.94 (13)
C2—C3—C4—C5	0.7 (3)	C1—S1—C10—C11	178.61 (12)
F1—C4—C5—C6	178.99 (17)	C15—C10—C11—C12	57.4 (2)
C3—C4—C5—C6	−0.6 (3)	S1—C10—C11—C12	−177.14 (13)
C4—C5—C6—C7	0.0 (3)	C10—C11—C12—C13	−56.3 (2)
C5—C6—C7—O1	−179.06 (17)	C11—C12—C13—C14	55.6 (2)
C5—C6—C7—C2	0.5 (3)	C12—C13—C14—C15	−55.5 (2)
C8—O1—C7—C6	179.06 (17)	C11—C10—C15—C14	−57.2 (2)
C8—O1—C7—C2	−0.56 (19)	S1—C10—C15—C14	179.30 (13)
C3—C2—C7—C6	−0.4 (3)	C13—C14—C15—C10	55.9 (2)
C1—C2—C7—C6	−179.45 (17)		

### Hydrogen-bond geometry (Å, °)

**Table:**

*D*—H···*A*	*D*—H	H···*A*	*D*···*A*	*D*—H···*A*
C6—H6···O2^i^	0.95	2.44	3.306 (2)	151
C10—H10···O3^ii^	1.00	2.33	3.274 (2)	157

Symmetry codes: (i) *x*, *y*, *z*−1; (ii) *x*−1, *y*, *z*.
